# Supplementary material for: Externalities in wild pig damages on U.S. crop and livestock farms: The role of landowner actions and landscape heterogeneity
Source: PLoS One. 2025 Apr 10;20(4):e0320316. doi: 10.1371/journal.pone.0320316 (PMC11984977; doi:10.1371/journal.pone.0320316)
Supplement: S1 File — Supporting Information. S1 Fig. Counties with Farm Bill-Funded Feral Swine Eradication and Control Pilot Programs (FSECPP) and those adjacent to FSECPP counties. Source: [25]. S2 Fig. Survey questions related to actions that attract wild animals on the respondent’s property and on surrounding properties. (Source: [10,11]). S3 Fig. Survey questions related to wild pig damages on pasture. (Source: [10]). S4 Fig. Survey questions related to wild pig damages to cropland. (Source: [11]). S5 Fig. Survey questions related to wild pig damages to other property. (Source: [10,11]). S1 Table. Summary of 14 Logistic regression models evaluated. S2 Table. Description of the USDA NASS survey questions used to construct the study variables. S3 Table. Summary Statistics (Farm Bill Counties and Adjacent). S4 Table. Marginal Effects of Determinants of Feral Pig Presence and Damage to Crop Operations for Farm Bill and Farm Bill Adjacent Counties. S5 Table. Marginal Effects of Determinants of Feral Pig Presence and Damage to Livestock Operations for Farm Bill and Farm Bill Adjacent Counties. (DOCX) [file pone.0320316.s001.docx]

**Supporting Information (SI) File**

*Externalities in wild pig damages on crop and livestock farms: neighboring landowner actions and the probability of pig presence and economic loss*

Submitted for consideration to be published in PLOS One

This Supporting Information (SI) file contains ancillary tables and figures to the main text of the article “Externalities in wild pig damages on crop and livestock farms: neighboring landowner actions and the probability of pig presence and economic loss” submitted for consideration to be published in PLOS One. The tables and figures are organized in order of their first mention in the main article.


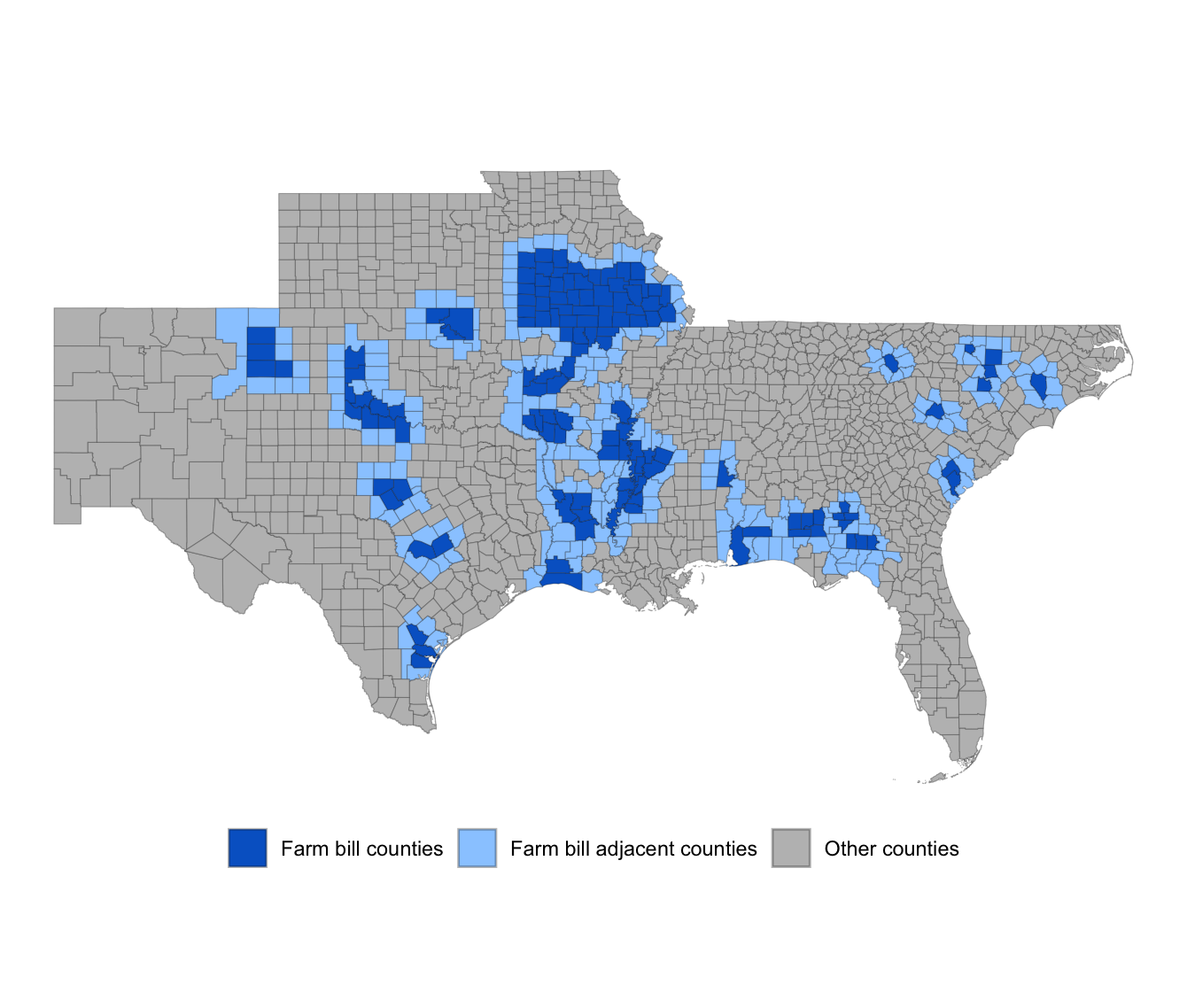


**Figure S1. Counties with Farm Bill-Funded Feral Swine Eradication and Control Pilot Programs (FSECPP) and those adjacent to FSECPP counties. Source: [24].**

Table S1 summarizes the dependent variables, operation locations, and operator types considered in each of the 14 logistic regression models that we estimated. The first column indicates the dependent variable. For instance, four models (Models 1, 5, 8, and 12) used ‘pig presence’ as the dependent variable. A total of seven regression models (Models 1 to 7) were estimated using data for all counties in our study area. Another seven models with identical variables (Models 8 to 14) were also estimated using data for only FSECPP counties and FSECPP-adjacent counties. The remaining columns indicate the data source (i.e., crop producer or livestock producer survey), the type of damage—which consisted of direct damages to crops only (Model 4), direct damages to pasture only (Model 11), or any crop, pasture, or other property damage (remaining models)--and the unit of data collection (i.e., crop parcel, livestock pasture, or whole farm operation).

| **Table S1.** Summary of 14 Logistic regression models evaluated | | | | | | | | | |
| --- | --- | --- | --- | --- | --- | --- | --- | --- | --- |
| Dependent  variable | All  Counties | | | |  | Farm Bill counties  + adjacent counties | | | |
|  | Crop survey | | Livestock survey | |  | Crop survey | | Livestock survey | |
|  | Property | Crop | | Property |  | Property | Field | | Property |
| Pig Presence | (1) |  | | (5) |  | (8) |  | | (12) |
| Pig Crop Damage | (2) | (4) | |  |  | (9) | (11) | |  |
| Pig Pasture Damage |  |  | | (6) |  |  |  | | (13) |
| Pig Any  Damage | (3) |  | | (7) |  | (10) |  | | (14) |

Figures S2 to S6 provide replications of the specific survey questions used to construct our study variables. The figure captions provide more detail on how each question was used.


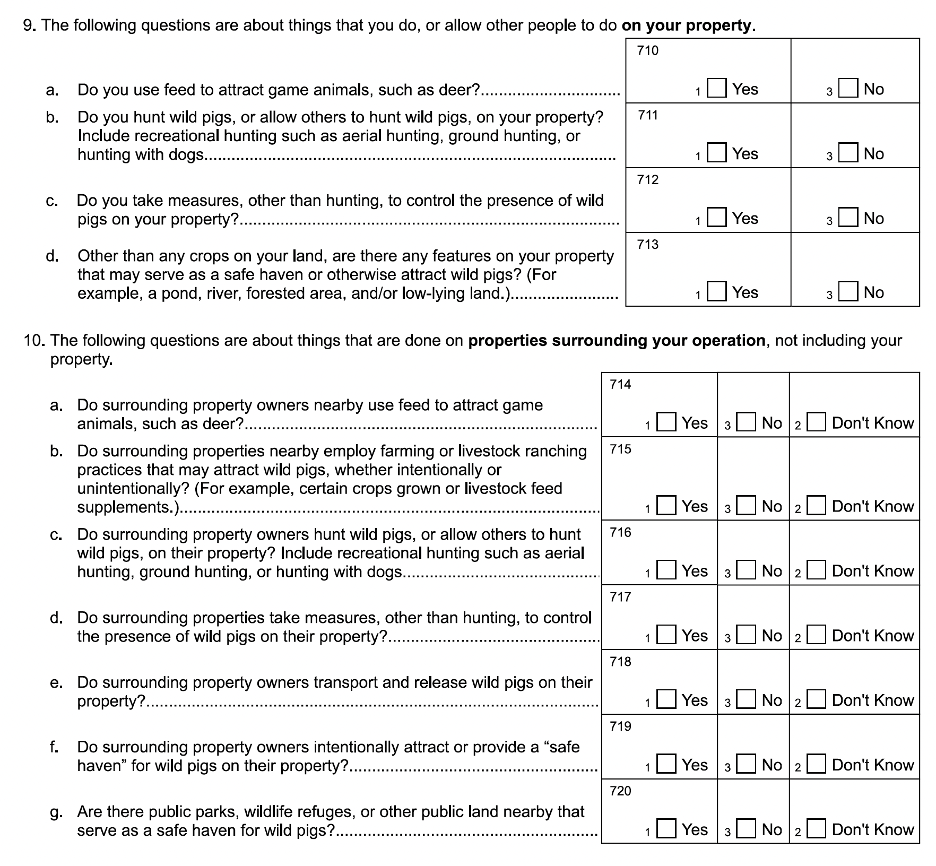


**Figure S2. Survey questions related to actions that attract wild animals on the respondent’s property and on surrounding properties (Source: [10-11]).**


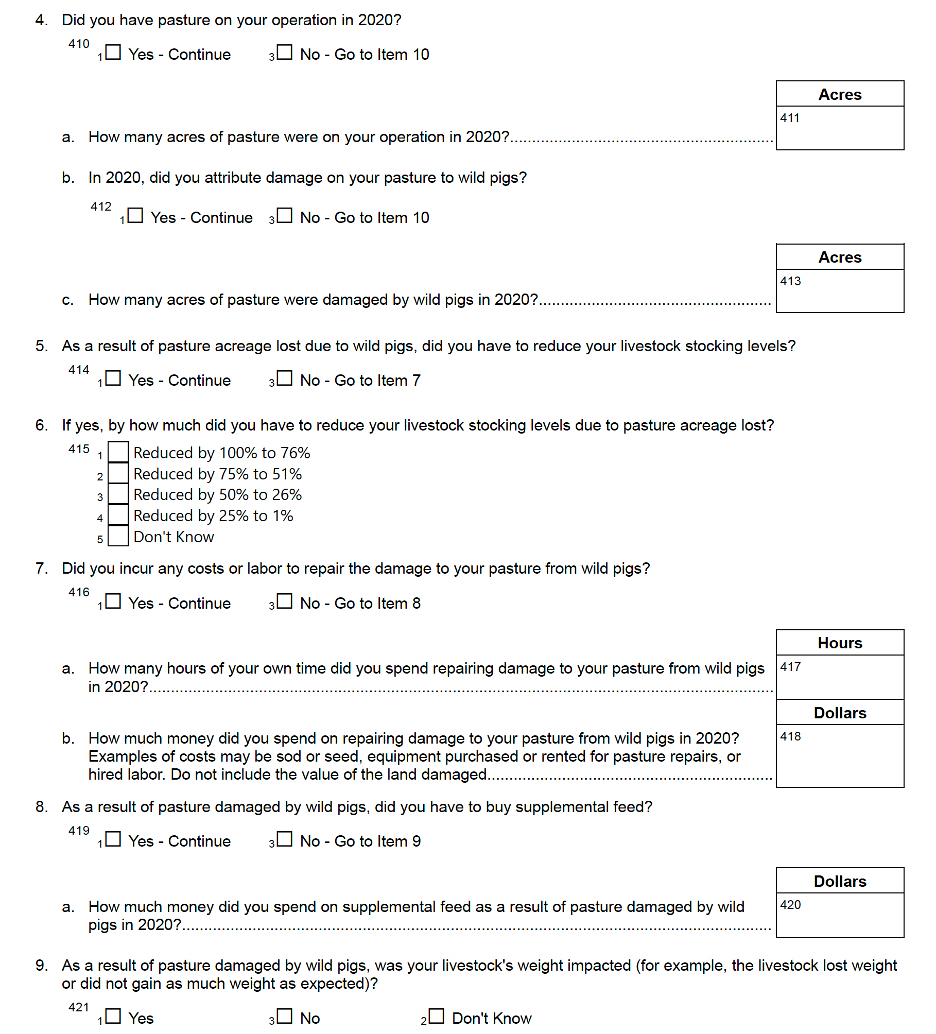


**Figure S3. Survey questions related to wild pig damages on pasture (Source: [10])**


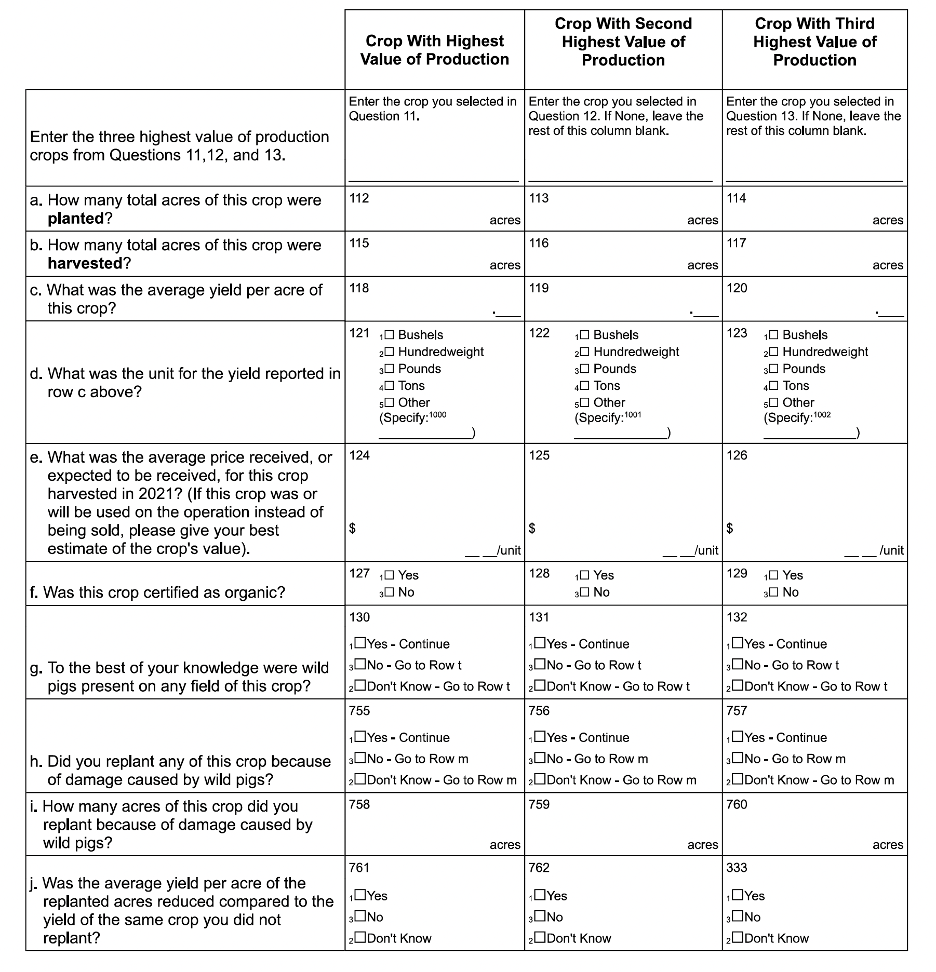


**Figure S4. Survey questions related to wild pig damages to cropland (Source: [11]).**


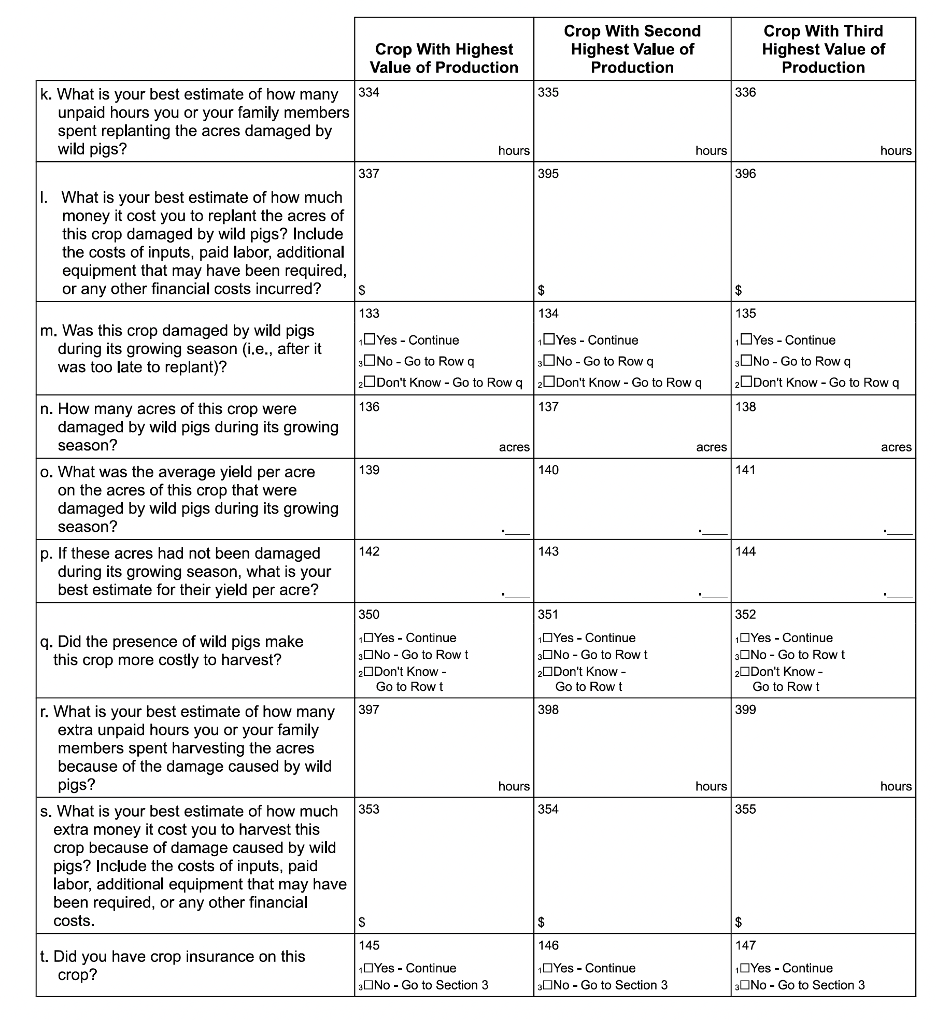


**Figure S4 (continued)**


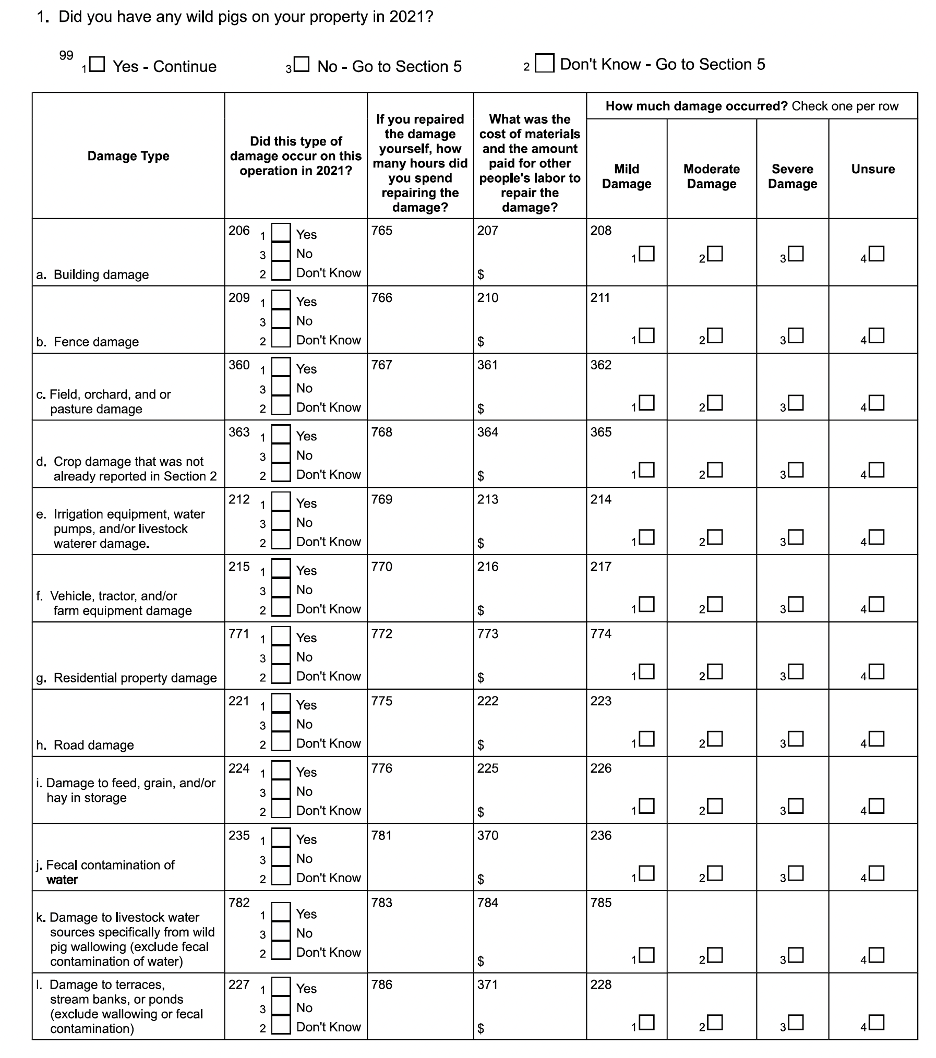


**Figure S5. Survey questions related to wild pig damages to other property (Source: [10-11]).**

The table below describes the specific survey questions that were used in the construction of the study variables. The

| **Table S2. Description of the USDA NASS survey questions used to construct the study variables.** | | | | | | |
| --- | --- | --- | --- | --- | --- | --- |
|  | Study variable | 2022 Crop Survey  Question Code | | 2021 Livestock Survey Question Code | |  |
| Wild Pig Presence and Damage | | |  | |  | |
|  | Pig Presence | ic99 | | ic99 | |  |
|  | Pig Damage to Crops or Pasture | ic755 OR ic133 OR ic350 | | Ic412 | |  |
| Attraction Actions (Landowner and/or neighbor) | | | | |  | |
|  | Landowner Attracts Game Animals | ic710 | | ic109 | |  |
|  | Neighbor Attracts Wildlife | ic714 OR ic716 AND NOT ic717 OR ic718 OR ic719 | | ic151 OR ic153 OR ic156 OR ic155 OR ic156 AND NOT ic154 | |  |
| Landowner Property Characteristics | | |  | |  | |
|  | Haven for Wild Pig | ic713 | | ic150 | |  |
|  | Large Farm | ic103 | | ic103 | |  |
|  | Crop or Pasture Acres (100s of acres) | ic112/100 | | ic411/100 | |  |
|  | Beef Farm |  | | ic400 | |  |
|  | Dairy Farm |  | | ic401 | |  |
|  | Hog Farm |  | | ic110 | |  |
|  | Sheep or Goat Farm |  | | ic111 OR ic112 | |  |
|  | Corn | ic750=1 OR ic751=1 OR ic752=1 | | | |  |
|  | Soybeans | ic750=2 OR ic751=2 OR ic752=2 | | | |  |
|  | Wheat | ic750=3 OR ic751=3 OR ic752=3 | | | |  |
|  | Rice | ic750=4 OR ic751=4 OR ic752=4 | | | |  |
|  | Sorghum | ic750=5 OR ic751=5 OR ic752=5 | | | |  |
|  | Peanuts | ic750=6 OR ic751=6 OR ic752=6 | | | |  |
| Neighboring Property Characteristics | | |  | |  | |
|  | Public Park, Wildlife Refuge, or Other Public Land Nearby | ic720 | | ic157 | |  |
|  | Neighbor Farms | ic715 | | ic152 | |  |

Note: Survey questions with three-level survey response formats (“Yes”, “Unsure”, “No”) were collapsed into dichotomous variables (“Yes”, “Other”) when constructing the study variables. The crop survey is described in [11] and the livestock survey is described in [10].

Tables S3 to S5 present summary statistics and marginal effects, subset to Farm Bill Counties and Adjacent

**Table S3. Summary Statistics (Farm Bill Counties and Adjacent).**

| VARIABLES | | Crop Farms | | Crop Farms (Parcel) | | Livestock Farms | |
| --- | --- | --- | --- | --- | --- | --- | --- |
|  |  | Mean | Std. Error | Mean | Std. Error | Mean | Std. Error |
| Wild Pig Presence and Damage | |  |  |  |  |  |  |
|  | Pig Presence | 0.446 | 0.027 |  |  | 0.441 | 0.031 |
|  | Pig Damage to Crops or Pasture | 0.340 | 0.026 |  |  | 0.346 | 0.031 |
|  | Pig Damage to Any Property | 0.395 | 0.025 |  |  | 0.414 | 0.031 |
|  | Pig Damage to Crops (Field Level) |  |  | 0.269 | 0.018 |  |  |
| Attraction Actions (Landowner and/or neighbor) | | |  |  |  |  |  |
|  | Landowner Attracts Game Animals | 0.381 | 0.029 | 0.379 | 0.027 | 0.312 | 0.029 |
|  | Neghbor Attracts Wildlife | 0.660 | 0.027 | 0.655 | 0.026 | 0.377 | 0.030 |
| Landowner Property Characteristics | |  |  |  |  |  |  |
|  | Haven for Wild Pig | 0.632 | 0.028 | 0.636 | 0.027 | 0.619 | 0.031 |
|  | Large Farm | 0.313 | 0.020 | 0.351 | 0.021 | 0.422 | 0.031 |
|  | Crop or Pasture Acres (100s of acres) | 8.387 | 0.496 | 5.064 | 0.270 | 2.681 | 0.229 |
|  | Beef Farm |  |  |  |  | 0.867 | 0.024 |
|  | Dairy Farm |  |  |  |  | 0.033 | 0.010 |
|  | Hog Farm |  |  |  |  | 0.049 | 0.014 |
|  | Sheep of Goat Farm |  |  |  |  | 0.017 | 0.004 |
|  | Corn |  |  | 0.341 | 0.015 |  |  |
|  | Soybeans |  |  | 0.329 | 0.016 |  |  |
|  | Wheat |  |  | 0.143 | 0.011 |  |  |
|  | Rice |  |  | 0.044 | 0.005 |  |  |
|  | Sorghum |  |  | 0.054 | 0.010 |  |  |
|  | Peanuts |  |  | 0.088 | 0.009 |  |  |
| Neighboring Property Characteristics | |  |  |  |  |  |  |
|  | Public Park, Wildlife Refuge, or Other Public Land Nearby | 0.233 | 0.025 | 0.254 | 0.026 | 0.113 | 0.017 |
|  | Neighbor Farms | 0.508 | 0.029 | 0.508 | 0.028 | 0.377 | 0.029 |
| Landscape Characteristics | |  |  |  |  |  |  |
|  | Carrying Capacity Low | 0.061 | 0.011 | 0.069 | 0.014 | 0.020 | 0.008 |
|  | Carrying Capacity Medium | 0.764 | 0.022 | 0.762 | 0.024 | 0.869 | 0.021 |
|  | Carrying Capacity High | 0.175 | 0.020 | 0.170 | 0.020 | 0.111 | 0.020 |
|  | Avg. Farm Size (100s of Acres) | 4.524 | 0.190 | 4.846 | 0.199 | 1.644 | 0.085 |
|  |  |  |  |  |  |  |  |

**Table S4: Marginal Effects of Determinants of Feral Pig Presence and Damage to Crop Operations for Farm Bill and Farm Bill Adjacent Counties.**

|  |  | Pig Presence | | Pig Crop Damage | | Pig Damage Any | | Pig Crop Damage Parcel | |
| --- | --- | --- | --- | --- | --- | --- | --- | --- | --- |
| VARIABLES | | dy/dx | Std. Error | dy/dx | Std. Error | dy/dx | Std. Error | dy/dx | Std. Error |
| Attraction Actions (Landowner and/or neighbor) | | |  |  |  |  |  |  |  |
|  | Landowner Attracts Game Animals | -0.043 | (0.042) | -0.012 | (0.035) | -0.008 | (0.041) | -0.014 | (0.027) |
|  | Neighbor Attracts Wildlife | 0.092* | (0.047) | 0.091** | (0.042) | 0.077 | (0.049) | 0.077** | (0.035) |
| Landowner Property Characteristics | |  |  |  |  |  |  |  |  |
|  | Haven for Wild Pig | 0.162*** | (0.042) | 0.07* | (0.042) | 0.13*** | (0.047) | 0.054* | (0.032) |
|  | Large Farm | 0.091** | (0.041) | 0.121*** | (0.036) | 0.106*** | (0.04) | 0.082*** | (0.03) |
|  | Crop or Pasture Acres (100s of acres) | 0.002 | (0.001) | 0.002** | (0.001) | 0.002* | (0.001) | 0.004*** | (0.001) |
|  | Soybeans |  |  |  |  |  |  | -0.058** | (0.025) |
|  | Wheat |  |  |  |  |  |  | -0.01 | (0.035) |
|  | Rice |  |  |  |  |  |  | -0.063 | (0.042) |
|  | Sorghum |  |  |  |  |  |  | 0.097 | (0.061) |
|  | Peanuts |  |  |  |  |  |  | 0.145*** | (0.038) |
| Neighboring Property Characteristics | |  |  |  |  |  |  |  |  |
|  | Public Park, Wildlife Refuge, or Other Public Land Nearby | 0.114*** | (0.043) | 0.067* | (0.036) | 0.043 | (0.041) | 0.041 | (0.028) |
|  | Neighbor Farms | 0.093** | (0.041) | 0.135*** | (0.033) | 0.134*** | (0.039) | 0.113*** | (0.028) |
| Landscape Characteristics | |  |  |  |  |  |  |  |  |
|  | Carrying Capacity Medium | 0.178*** | (0.068) | 0.193*** | (0.058) | 0.209*** | (0.064) | 0.18*** | (0.038) |
|  | Carrying Capacity High | 0.228*** | (0.088) | 0.219*** | (0.071) | 0.265*** | (0.081) | 0.18*** | (0.038) |
|  | Avg. Farm Size (100s of Acres) | -0.001 | (0.005) | 0 | (0.005) | -0.004 | (0.005) | -0.003 | (0.004) |
| State Fixed Effects | | Yes |  | Yes |  | Yes |  | Yes |  |
| Observations | | 1,030 |  | 1,030 |  | 1,030 |  | 1,916 |  |
| Pseudo R2 | | 0.537 |  | 0.487 |  | 0.507 |  | 0.437 |  |

Notes: All marginal effects are average marginal effects.

**Table S5. Marginal Effects of Determinants of Feral Pig Presence and Damage to Livestock Operations for Farm Bill and Farm Bill Adjacent Counties.**

|  |  | Pig Presence | |  | Pig Crop Damage | |  | Pig Damage Any | |
| --- | --- | --- | --- | --- | --- | --- | --- | --- | --- |
| VARIABLES | | dy/dx | Std. Error |  | dy/dx | Std. Error |  | dy/dx | Std. Error |
| Attraction Actions (Landowner and/or neighbor) | |  |  |  |  |  |  |  |  |
|  | Landowner Attracts Game Animals | 0.124*** | (0.04) |  | 0.058 | (0.046) |  | 0.09** | (0.041) |
|  | Neighbor Attracts Wildlife | 0.037 | (0.047) |  | -0.024 | (0.044) |  | 0.006 | (0.049) |
| Landowner Property Characteristics | |  |  |  |  |  |  |  |  |
|  | Haven for Wild Pig | 0.166*** | (0.035) |  | 0.198*** | (0.043) |  | 0.219*** | (0.037) |
|  | Large Farm | 0.137*** | (0.037) |  | 0.102** | (0.045) |  | 0.142*** | (0.037) |
|  | Crop or Pasture Acres (100s of acres) | 0.001 | (0.003) |  | 0.001 | (0.001) |  | 0.001 | (0.001) |
|  | Beef Farm | 0.065 | (0.075) |  | 0.086 | (0.083) |  | 0.052 | (0.078) |
|  | Dairy Farm | 0.016 | (0.058) |  | 0.087 | (0.063) |  | 0.008 | (0.062) |
|  | Hog Farm | -0.034 | (0.045) |  | -0.111** | (0.052) |  | -0.046 | (0.048) |
|  | Sheep or Goat Farm | 0.059 | (0.064) |  | 0.083 | (0.078) |  | 0.062 | (0.067) |
| Neighboring Property Characteristics | |  |  |  |  |  |  |  |  |
|  | Public Park, Wildlife Refuge, or Other Public Land Nearby | 0.075 | (0.055) |  | 0.023 | (0.071) |  | 0.083 | (0.057) |
|  | Neighbor Farms | 0.086* | (0.049) |  | 0.078 | (0.048) |  | 0.067 | (0.049) |
| Landscape Characteristics | |  |  |  |  |  |  |  |  |
|  | Carrying Capacity Medium | 0.312*** | (0.078) |  | 0.176* | (0.095) |  | 0.288*** | (0.077) |
|  | Carrying Capacity High | 0.277** | (0.111) |  | 0.23** | (0.107) |  | 0.269** | (0.117) |
|  | Avg. Farm Size (100s of Acres) | -0.005 | (0.01) |  | -0.01 | (0.011) |  | -0.004 | (0.009) |
|  |  |  |  |  |  |  |  |  |  |
| State Fixed Effects | | Yes |  |  | Yes |  |  | Yes |  |
|  |  |  |  |  |  |  |  |  |  |
| Observations | | 1,404 |  |  | 1,404 |  |  | 1,404 |  |
| Pseudo R2 | | 0.630 |  |  | 0.520 |  |  | 0.6 |  |

Notes: All marginal effects are average marginal effects
