# Supplementary material for: Externalities in wild pig damages on U.S. crop and livestock farms: The role of landowner actions and landscape heterogeneity
Source: PLoS One. 2025 Apr 10;20(4):e0320316. doi: 10.1371/journal.pone.0320316 (PMC11984977; doi:10.1371/journal.pone.0320316)
Supplement: Attachment — Submitted filename: Response to Reviewers - PONE-D-24-51915.docx [file pone.0320316.s003.docx]

**Response to Reviewer 1**

**First Revision**

**Manuscript ID PONE-D-24-51915**

Note: The page numbers cited refer to the version of the revised manuscript without track changes.

1. **Introduction part line 105; you add your results in the inappropriate place; please paraphrase or transfer to the results part.**

*Response: Thank you for taking time to review the paper, sharing your interest in the topic, and providing your comments. We deleted the text that previewed our results in the introduction.*

2. **How did you analyze your collected data? It should be clear and simple for understanding. Lacks clarity your method of analysis.**

*Response: Thank you for noting that our methods section did not provide a rationale for using a logistic regression framework. To clarify this, we added the following sentence to the first paragraph of the Model section (lines 127-132): “Logistic regression models help understand the influence of potential determinants on the likelihood of an occurrence (i.e., pig presence or damages) by calculating the probability of a binary outcome (yes/no) based on a set of explanatory variables, where each variable's coefficient indicates how much that variable increases or decreases the odds of the occurrence happening, indicating which have the most significant impact on the predicted outcome”*

3. **There is no consistency in using % or percentile; please use either of these throughout your text body.**

*Response: Thank you for raising this issue. We revised the text to ensure consistent usage of these terms in our reporting. The revised manuscript now only refers to percentage point changes. We eliminated all mention of percentile. We also clarified this in the revised manuscript, in the first paragraph of the results section. The revised text now reads (lines 284-287):*

*“For ease of interpretation, we report only the average marginal effects for all models, which represent the estimated change in the likelihood of being impacted by wild pigs and are interpreted as percentage-point increases and decreases in the probability of being impacted by wild pigs.”*

*Note that we still use the percent symbol (%) in our discussion of survey summary statistics, however we believe the above clarification should help readers more easily follow and interpret our findings.*

4. **In tables, there is dy/dx; this symbol/formula, needs what it shows in the table captions!**

*Response: We thank the reviewer for pointing this out. We added footnotes to both Tables 2 and 3 explaining that dy/dx represent marginal effects (i.e. derivatives) with respect to each of the explanatory variables.*

5. **There is no discussion part, which is basic to relate your findings with other scholars?**

*Response: We made two changes to better incorporate a discussion element into our revised manuscript. First, we created a new stand-alone discussion section which appears after the results section. In the new discussion section, we highlight the practical meaning of our main results. Second, within the new discussion section, we expanded our discussion points to reflect how they relate to other previous literature. The newly integrated literature citations have been added to our reference list.*

**Response to Reviewer 2**

**First Revision**

**Manuscript ID PONE-D-24-51915**

Note: The page numbers cited refer to the version of the revised manuscript without track changes.

1. **Clarification of Citations: On page 4, line 91, the paper references "research" without specifying which studies are cited. Providing detailed citations in such instances would bolster the paper’s credibility and situate its findings within the broader body of literature.**

*Response: Thank you for taking time to review the paper, sharing your interest in the topic, and providing your comments. We agree with your suggestions and have added three references to studies illustrating such research: Epanchin‐Niell et al.* [*2010*](https://www.ncbi.nlm.nih.gov/pmc/articles/PMC6850443/#cobi13266-bib-0014)*; Graham* [*2014*](https://www.ncbi.nlm.nih.gov/pmc/articles/PMC6850443/#cobi13266-bib-0018)*; Ervin & Frisvold* [*2016*](https://www.ncbi.nlm.nih.gov/pmc/articles/PMC6850443/#cobi13266-bib-0015)*.*

2. **Introduction of Key Concepts: The concept of wild pigs as res nullius animals (not owned by any individual) is introduced relatively late in the paper, on page 7. Including this concept in the introduction would provide essential context and help frame the issue for readers early in the discussion.**

*Response: Thank you for raising this point and introducing the res nullius terminology. The concept of res nullius (Latin for "nobody's property") refers to things that are not owned by anyone and are available for acquisition, such as wild animals or unowned land under certain legal systems. However, feral swine in the United States occupy a unique legal status that doesn't perfectly align with traditional Roman law classifications like res nullius or res publicae (public property). Feral swine are best classified as a regulated invasive species or nuisance wildlife. Their legal treatment reflects a focus on harm reduction and management rather than ownership, fitting neither res nullius nor res publicae frameworks precisely.*

*However, we agree that the characteristics of feral swine that make them align closely with that of res nullius (mobility, lack of geographical nor jurisdictional boundaries) should be introduced earlier in the manuscript. We have therefore (1) added the following sentence at the beginning of the introduction (lines 53-54): “Invasive species are mobile, have multiple vectors, and ignore property, jurisdictional, and tenure boundaries [1]” and (2) reorganized the introduction to lay out the complex challenges posed by the spatial externalities created by the presence of invasive species in general, and of wild pigs in particular.*

*We also address this comment, in part, in our response to comment #3 below that introduces a global perspective.*

3. **Global Perspective: While the paper focuses on the southeastern United States, it misses an opportunity to connect its findings to similar challenges faced in other parts of the world. Human-wildlife conflicts involving res nullius animals are a global issue, and a brief reference to analogous situations in other regions could broaden the paper’s applicability. Comparative research, even in summary form, would highlight shared challenges and potential cross-contextual solutions, enriching the discussion.**

*Response: We agree this is a limitation to our study and also believe that a future study of related challenges from a global perspective would make a valuable contribution to the literature. We incorporated a discussion of this observation as a limitation to our study in the new “Discussion” section of the manuscript. The revised text now reads (lines 469-475),*

*“Finally, our study’s geographical scope was limited to the continental United States. However, analogous issues exist around the world, with other regions also experiencing problems related to crop and livestock damage caused by wild animals (e.g., Rao et al., 2002; Govind and Jayson, 2018; Alemayehu and Tekalign, 2022). Future research that combines insights and evidence from previous studies on this topic from a global perspective would provide a valuable contribution to the literature by highlighting common challenges and potential solutions that could be applied across different contexts.”*
